# Hexokinase as a Central Hub in Neurodegeneration: From Metabolic Dysfunction to Therapeutic Innovation

**DOI:** 10.14336/AD.2025.0891

**Published:** 2026-09-27

**Authors:** Shiyuan Cai, Yanjun Liu, Bo Liu, Hongli Liao, Ke Li

**Affiliations:** ^1^Department of Pharmacy, The People's Hospital of Miyi County, Panzhihua 617000, Sichuan, China.; ^2^Key Laboratory of Structure-Specific Small Molecule Drugs at Chengdu Medical College of Sichuan Province, School of Pharmacy, Chengdu Medical College, Chengdu.610500, Sichuan, China.; ^3^Department of Urology, The People's Hospital of Miyi County, Panzhihua 617000, Sichuan, China.

**Keywords:** hexokinase, neurodegeneration, metabolic reprogramming, mitochondrial dysfunction, therapeutic targets

## Abstract

Neurodegenerative diseases represent an escalating global health crisis affecting more than 55 million people worldwide; however, underlying mechanisms remain unclear, and therapeutic breakthroughs are elusive. Emerging evidence indicates that hexokinase (HK), the rate-limiting glycolytic enzyme, functions as a master regulator orchestrating neuronal survival through metabolic-mitochondrial coupling. This review consolidates emerging paradigms revealing that HK maintains neuronal viability through its obligate interaction with mitochondrial VDAC1, forming a metabolic checkpoint that integrates energy status with survival signaling. Disease-specific HK dysfunction patterns precede clinical manifestations and drive pathological cascades across primary neurodegenerative conditions. Pathological proteins characteristic of neurodegeneration—amyloid-β in AD, α-synuclein in PD, mutant SOD1 in ALS, and huntingtin in HD—converge to disrupt the HK-VDAC1 axis through distinct molecular mechanisms, triggering mitochondrial permeabilization, bioenergetic collapse, and inflammatory activation. This uncoupling event promotes VDAC1 oligomerization, enabling the cytosolic release of mtDNA, which in turn activates the NLRP3 inflammasome while depleting antioxidant capacity, establishing self-perpetuating neuroinflammatory cycles. The literature reveals that HK functions as a molecular rheostat, determining neuronal fate through glucose-6-phosphate-mediated feedback control, modulation of growth factor signaling, and regulation of apoptosis/survival pathways. Therapeutic targeting of HK through peptide interventions, enzymatic modulation, and gene therapy demonstrates robust neuroprotective effects across multiple disease models. Meanwhile, combination strategies addressing metabolic-inflammatory networks show synergistic efficacy. These insights position HK as a convergent therapeutic nexus offering unprecedented opportunities for precision intervention in neurodegeneration, with potential for early diagnostic applications and preventive strategies that could transform treatment paradigms for conditions affecting millions worldwide.

## 1. Introduction

Neurodegenerative diseases (NDs) represent one of the most formidable health challenges of the 21st century, with their incidence increasing dramatically as the global population ages [1, 2]. Conditions such as Alzheimer’s disease (AD), Parkinson’s disease (PD), and amyotrophic lateral sclerosis (ALS) impose devastating burdens not only on patients and their families but also on healthcare systems worldwide [2]. According to the World Health Organization, approximately 55 million people globally have dementia, with projections indicating that this figure will reach 139 million by 2050 [3]. PD affects more than 10 million individuals worldwide [4], whereas ALS, although relatively rare, is among the most challenging neurological disorders because of its rapid progression [5]. Despite decades of intensive scientific effort, therapeutic options for these diseases remain severely limited, with existing medications focused primarily on symptom management rather than effectively halting or reversing disease progression. Traditional single-target drug development strategies have repeatedly encountered setbacks, creating an urgent need to explore novel therapeutic avenues from entirely new perspectives [6, 7]. In recent years, increasing evidence has demonstrated that energy metabolism dysregulation constitutes a central pathological hallmark of NDs, a discovery that has opened new therapeutic avenues for disease management. As the most energy-demanding organ in the human body, the brain consumes approximately 20% of the total glucose and oxygen, rendering neurons highly vulnerable to energy supply disruptions; consequently, any metabolic perturbation can precipitate neuronal dysfunction [8-10]. In AD, cerebral glucose hypometabolism frequently precedes the clinical onset of symptoms by 5-10 years, providing crucial insights for early disease diagnosis [11]. Dopaminergic neurons in PD patients exhibit distinctive metabolic reprogramming phenomena characterized by a shift from oxidative phosphorylation to glycolysis—a metabolic transition intimately linked to neuronal survival [12]. Patients with ALS manifest motor neuron-specific energy crises, where mitochondrial dysfunction and insufficient ATP production serve as critical drivers of disease progression [13, 14]. These findings suggest that modulating cellular metabolic states, particularly through enhancing glycolytic pathway activity, may offer novel neuroprotective therapeutic strategies [15, 16]. Metabolic reprogramming not only ameliorates the energy supply status of neurons but also may exert comprehensive neuroprotective effects through the regulation of oxidative stress, inflammatory responses, and cell death pathways [15]. These metabolic alterations highlight the potential of targeting key glycolytic enzymes as a therapeutic strategy [16].

Among the numerous enzymes in the glycolytic pathway, hexokinase (HK), the first rate-limiting enzyme that catalyzes glucose phosphorylation, is of great importance [17]. HK not only governs glycolytic flux but also has multiple biological functions, including the regulation of cellular energy metabolism, maintenance of mitochondrial functional stability, and inhibition of apoptosis [17]. The HK family comprises four major isoforms, with HK1 and HK2 being most abundantly expressed in the nervous system [18, 19]. These isoforms form functional complexes by binding to voltage-dependent anion channels on the mitochondrial outer membrane, thereby ensuring efficient adenosine triphosphate (ATP) utilization while preventing the opening of the mitochondrial permeability transition pore (MPTP) and exerting antiapoptotic effects [18, 20]. More importantly, the HK product glucose-6-phosphate serves not only as a glycolytic intermediate but also as the initial substrate for the pentose phosphate pathway, providing support for nicotinamide adenine dinucleotide phosphate (NADPH) generation and antioxidant defense mechanisms [21]. This multifunctional capacity positions HK as a critical nexus linking energy metabolism, oxidative stress, and cell fate determination, conferring unique advantages as a therapeutic target [18]. Compared with conventional single-function targets, modulating HK activity can influence multiple pathological processes simultaneously, resulting in more comprehensive and effective neuroprotection [22, 23].

As the understanding of neurodegenerative disease mechanisms has deepened, the role of HK in these disorders has garnered increasing attention. Accumulating evidence reveals significant alterations in HK expression and activity within AD brain tissues, with characteristic temporal patterns emerging throughout disease progression [11, 24]. In PD, the aggregation of α-synuclein (α-syn) promotes the detachment of HK from mitochondria, thereby impairing its protective functions. Conversely, interventions that stabilize this HK-mitochondrial linkage have been shown to exert significant neuroprotective effects [25]. In ALS, superoxide dismutase 1 (SOD1) mutations directly impair HK function, resulting in energy metabolism dysfunction in motor neurons [13, 14]. These findings not only illuminate the critical role of HK dysfunction in disease pathogenesis but also provide a theoretical foundation for HK-targeted therapeutic strategies. In recent years, several studies have explored neuroprotective therapeutic approaches through enhancing HK activity, stabilizing its mitochondrial localization, or mimicking its functions, with preliminary results demonstrating promising therapeutic potential [15, 18, 22, 26].

However, current research still faces numerous limitations and challenges. First, most studies remain confined to mechanistic exploration, with a substantial gap existing between basic research and clinical application. While we have gained considerable insight into the mechanisms by which HKs function in NDs, translating this knowledge into effective therapeutic interventions remains a formidable challenge [16]. Second, HK expression and function vary across different tissues and cell types, making it a significant technical challenge to achieve nervous system-specific modulation while avoiding adverse effects on other systems [16]. Furthermore, HK regulation involves complex signalling networks, and simply enhancing HK activity may produce unexpected side effects, necessitating more refined and individualized regulatory strategies. Finally, the translational process from laboratory research to clinical trials requires the resolution of a series of technical issues, including drug delivery, targeting specificity, and safety assessment.

Based on the background as mentioned above, this review aims to systematically elucidate the mechanisms of HK action in NDs and its therapeutic potential, providing a comprehensive theoretical foundation for further research and clinical translation in this field. This review presents an in-depth analysis of the specific mechanisms by which HK functions in AD, PD, ALS, and other disorders from multiple perspectives, including energy metabolic imbalance, mitochondrial dysfunction, the neuroinflammation-oxidative stress axis, and neuronal fatedetermination. By integrating the latest research advances, this review reveals how HK dysfunction participates in disease onset and progression through its effects on cellular metabolism, mitochondrial function, inflammatory responses, and cell death pathways while exploring the feasibility and challenges of HK-targeted therapeutic strategies.

**Figure 1.**
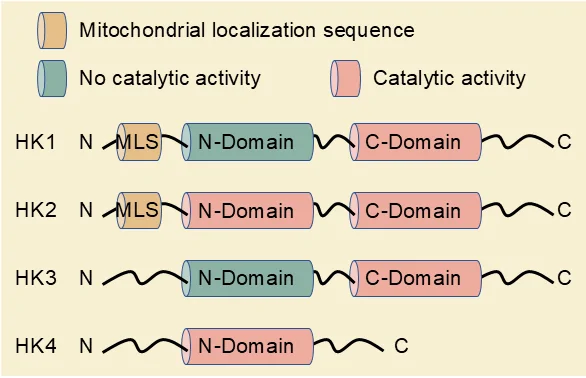
**Hexokinase Domain Architecture and Mitochondrial Targeting**. Detailed structural organization of hexokinase showing the N-terminal and C-terminal domains with the mitochondrial localization signal (MLS). The N-domain contains the mitochondrial-binding region essential for VDAC1 interaction, while the C-domain houses the catalytic site. This domain architecture enables HK to function as a metabolic sensor at the mitochondrial outer membrane, facilitating the integration of glycolytic flux with mitochondrial bioenergetics in neuronal cells.

## 2. HK and metabolic reprogramming in NDs

### 2.1 Molecular characteristics and isoform functions of HK

HK, as the first rate-limiting enzyme in the glycolytic pathway, plays a pivotal regulatory role in neuronal energy metabolism. The HK family comprises four major isoforms (HK1-4), with HK1 and HK2 being most abundantly expressed in the nervous system [18, 19]. As shown in Figure 1, hexokinases contain distinct N-terminal and C-terminal domains, with the N-domain harboring the mitochondrial localization sequence (MLS) that facilitates their targeting to the outer mitochondrial membrane. This structural organization is critical for establishing the metabolic coupling between glycolysis and oxidative phosphorylation. Proteomic analysis by Bert C. Lynn et al. revealed that HK1 exhibits an “HXH” (high-low-high) expression pattern during AD progression: significant upregulation in mild cognitive impairment and early AD stages, followed by a slight decline in late-stage AD, while remaining elevated compared with controls [27]. This temporal variation reflects the adaptive regulation of HK1 at different disease stages, suggesting its crucial role in maintaining neuronal energy homeostasis.

HK isoforms exhibit functional specificity and cell type specificity across different NDs. HK1 is localized primarily to the outer mitochondrial membrane, where it maintains mitochondrial functional stability through its interaction with voltage-dependent anion channel 1 (VDAC1) [18, 28]. Simon M. Bell et al. demonstrated that significantly reduced HK1 activity in sporadic AD astrocytes leads to impaired ATP production capacity, decreased mitochondrial membrane potential, and weakened glycolytic reserve capacity [29]. This functional deficiency impairs both astrocytic metabolism and the capacity of astrocytes to provide metabolic support to neurons. In contrast, Juan F. Codocedo et al. reported that HK2 plays a crucial role in microglial inflammatory activation, with its expression levels showing a dose-dependent relationship with the intensity of the inflammatory response [30]. A moderate reduction in HK2 can suppress excessive microglial activation and reduce inflammatory factor production. At the same time, a complete deficiency impairs basic cellular functions, indicating the precise regulatory role of HK2 in maintaining microglial functional homeostasis [30].

The subcellular localization and functional specificity of different HK isoforms determine their distinct roles in NDs. HK1 is primarily responsible for maintaining basal metabolic demands and mitochondrial stability, and its functional abnormalities often lead to a neuronal energy crisis and cell death [31]. HK2 is involved in stress responses and inflammatory regulation, potentially playing a protective role in early disease stages, but prolonged activation may promote pathological progression [30, 32]. Temporal analysis revealed that VDAC1 similarly exhibits an HXH pattern of changes with disease progression, suggesting the synergistic role of the HK-VDAC1 complex in energy metabolism and mitochondrial dysfunction [27]. This coordinated pattern of changes reflects adaptive cellular responses when confronted with pathological stress, where early upregulation may represent compensatory mechanisms. In contrast, the relative decline in later stages may reflect cellular functional exhaustion [27]. These isoform-specific functions provide the foundation for understanding disease-specific metabolic alterations.

### 2.2 Disease-Specific Patterns of Metabolic Reprogramming

In addition to these molecular characteristics, different neurodegenerative diseases exhibit distinct patterns of HK-mediated metabolic reprogramming. Metabolic reprogramming in different NDs results in unique molecular characteristics and spatiotemporal distribution patterns. In AD, metabolic abnormalities manifest as early and progressive functional dysfunction with pronounced regional selectivity. Through enzymatic activity analysis, M. Bigl et al. reported that phosphofructokinase and pyruvate kinase activities were significantly increased in the frontal and temporal cortices, whereas HK activity changes were relatively mild [33]. This selective enzymatic activity alteration reflects AD-specific metabolic adaptation patterns. Such enzymatic imbalances may cause metabolic intermediate accumulation, subsequently disrupting cellular functions. Single-cell transcriptomic studies by William J. Morrey et al. further revealed that early metabolic changes are driven primarily by glutamate-aspartate transporter (GLAST^+^) astrocytes, with HK2 significantly upregulated in these cells, accounting for the observed metabolic hyperactivity phenomenon [34]. Longitudinal studies of HK activity in leukocytes revealed a progressive increase in HK activity during normal aging and AD progression, with these changes occurring in early disease stages, suggesting that HK activity may serve as a biomarker for disease progression [35]. Research by David L. Marcus et al. on the microvasculature of AD patients revealed significantly decreased HK activity in temporal lobe microvessels, accompanied by reduced glucose transporter expression, indicating the vital role of the vascular system in disease development [36]. These vascular metabolic abnormalities may exacerbate insufficient energy supply to brain tissue, creating a vicious cycle.

PD exhibits unique metabolic reprogramming characteristics, primarily manifesting in dopaminergic neuron-specific metabolic pattern transitions. Hojin Kang et al. reported that dopaminergic neurons undergo metabolic conversion from oxidative phosphorylation to glycolysis, with the Parkin-interacting substrate (PARIS) protein reprogramming glucose metabolism through the transcriptional suppression of transketolase and markedly elevated HK2 expression in the substantia nigra. Although this metabolic reprogramming can provide a rapid ATP supply for high-energy-demanding dopaminergic neurons, it also significantly reduces energy production efficiency, increasing the susceptibility of neurons to further metabolic stress [12]. San-Qiao Yang et al. further confirmed that this enhanced aerobic glycolysis (Warburg effect) can be regulated through the leptin-hydrogen sulfide signalling axis, providing protective effects for dopaminergic neurons [37]. Research by Amrita Datta Chaudhuri et al. revealed the protective mechanism of microRNA-7 in PD, which prevents MPTP-induced cell death by promoting glycolysis [38]. This regulatory mechanism highlights the critical role of post-transcriptional regulation in metabolic reprogramming. Through comparisons of different mouse strains and dosing regimens, Anjana Pathania et al. reported significant differences in the degree of mitochondrial dysfunction and energy metabolism impairment in MPTP-induced Parkinsonian syndrome, suggesting the influence of genetic background on metabolic reprogramming [39].

Metabolic reprogramming in ALS is characterized by significant regional and cell type specificity. In the SOD1-G93A model, Silvia Ravera et al. reported that glucose breakdown was substantially decreased in presynaptic regions of the spinal cord and motor cortex, whereas nonsynaptic regions presented compensatory enhancement, with HK activity notably increased during the presymptomatic phase [13]. This heterogeneous pattern may reflect the selective vulnerability of motor neurons, where presynaptic metabolic defects initially impair neural conduction before causing neuronal death. Research by Andrea Magrì et al. demonstrated that a small HK1 peptide could rescue SOD1-G93A-induced mitochondrial accumulation and ATP-related respiratory function, suggesting the crucial role of HK1 in maintaining motor neuron function [14].

Metabolic abnormalities in Huntington’s disease (HD) also exhibit unique characteristics. Arthur J.L. Cooper et al. reported that under metabolic stress conditions, HK activity was significantly increased in fibroblasts from HD patients, indicating compensatory activation of the glycolytic pathway [40]. ALBERTO ZANELLA et al. reported that red blood cells from HD patients exhibited metabolic disorders and membrane abnormalities, with considerably diminished glucose utilization accompanied by elevated levels of membrane lipid peroxidation [41]. These systemic metabolic abnormalities suggest that HD is not only a central nervous system disease but also involves systemic metabolic dysfunction.

### 2.3 Spatiotemporal characteristics of energy metabolic imbalance

Energy metabolism imbalances in NDs exhibit distinct spatiotemporal specificity, which reflects the pathogenic mechanisms and progression patterns of these diseases. Dynamic fluorodeoxyglucose positron emission tomography (FDG-PET) studies by Morand Piert et al. demonstrated that glucose metabolic rates were significantly reduced in the temporoparietal and precuneus regions of AD patients and that these changes highly overlapped with the spatial distribution of neuronal dysfunction and synaptic loss [11]. Importantly, these metabolic abnormalities often appear years before clinical symptoms, providing important biomarkers for early disease diagnosis. The reduction in glucose transport and phosphorylation determined by dynamic FDG-PET not only reflects abnormal HK function but also reveals coordinated dysfunction of the entire glucose metabolic pathway.

Different cell types exhibit differential sensitivities to energy metabolic imbalances, and this differential sensitivity may explain the phenomenon of selective neuronal loss in NDs. Research by William J Morrey et al. demonstrated that neurons and astrocytes in AD present distinctly different metabolic adaptation patterns: neurons show decreased glucose transporter (GLUT) 3 expression and reduced HK activity, reflecting direct impairment of their metabolic capacity, whereas astrocytes compensate by upregulating GLUT1 and HK expression, attempting to maintain the local energy supply [34]. This cell type-specific metabolic response may play a protective role in the early stages of disease, but as the disease progresses, the compensatory capacity of astrocytes gradually becomes exhausted, ultimately leading to functional failure of the entire neural network. In PD, dopaminergic neurons are susceptible to metabolic imbalance because of their unique firing patterns and high energy demands. Research by G. GILLE et al. revealed that these neurons exhibit greater vulnerability when facing oxidative stress, with their mitochondrial function being more susceptible to damage [42]. PASTORIS et al.’s study on MPTP-induced Parkinsonian syndrome revealed that skeletal muscle also exhibits biochemical abnormalities, suggesting that metabolic abnormalities in PD patients are not limited to the central nervous system but also involve energy metabolism in peripheral tissues [43].

Complex interaction networks exist between energy metabolic abnormalities and disease pathological processes, forming multiple positive feedback loops. Spatial transcriptomic analysis revealed that the decline in glucose metabolism observed in AD patients is spatially correlated with amyloid plaque deposition, with metabolically abnormal regions often overlapping with pathological protein aggregation areas. Research by William J Morrey et al. demonstrated that early metabolic changes occur before amyloid-beta (Aβ) deposition and tau pathology, suggesting that metabolic abnormalities may be primary events in disease rather than secondary changes [34]. Energy metabolic reprogramming in PD patients is often accompanied by abnormal accumulation of α-syn. Studies by Ziba Dehghani [25] and Megha Rajendran et al. [44] showed that α-syn aggregates can directly interfere with the mitochondrial localization of HK, forming a vicious cycle of pathological protein-metabolic abnormalities.

The temporal progression of metabolic abnormalities also reflects the characteristic features of different disease stages. In the early stages of disease, metabolic reprogramming may primarily manifest as compensatory adaptation, with cells maintaining the ATP supply by increasing glycolytic enzyme activity. Research by Simon M. Bell et al. demonstrated that increasing HK1 expression can significantly improve mitochondrial function and glycolytic activity in sporadic AD astrocytes, suggesting the therapeutic potential of early intervention [29]. However, as the disease progresses, sustained metabolic stress leads to gradual cellular dysfunction, failure of compensatory mechanisms, and ultimately irreversible neuronal damage. G. Liguri et al. studied energy metabolism-related enzymes in AD patients and reported significant alterations in the activity of key enzymes such as Na^+^/K^+^-ATPase and Ca^2+^-ATPase, reflecting abnormalities in cellular membrane ion pump function [45]. This abnormality not only affects basic cellular physiological functions but also exacerbates energy consumption, resulting in a vicious cycle of energy crisis. Research by Yury G. Kaminsky et al. on erythrocyte 2,3-diphosphoglycerate metabolism revealed age-related metabolic defects, suggesting that the general decline in metabolic function during aging may increase susceptibility to NDs [46].

In summary, HK-mediated metabolic reprogramming in NDs exhibits remarkable complexity and specificity across disease types, cell types, and disease stages. This complexity reflects the precision of neural metabolic regulation and provides crucial insights for understanding disease pathogenesis. Elucidating these HK-dependent mechanisms offers new foundations for early diagnosis, disease monitoring, and precision therapeutic strategies in NDs.

## 3. HK-Mediated Mitochondrial Functional Regulatory Network

### 3.1 Structural basis of the HK-VDAC1 interaction

The functional interaction between HK and mitochondria is achieved primarily through its binding to VDAC1 on the mitochondrial outer membrane. Structural biology research [28] reveals that VDAC1 possesses a barrel-like structure composed of 19 β-strands, with an N-terminal α-helix located on the inner wall of the channel that participates in voltage sensing. As a mitochondrial “gatekeeper” protein, VDAC1 not only regulates membrane permeability but also participates in regulating apoptosis through interactions with various proteins, including HK. This unique structural feature enables VDAC1 to exert precise regulatory functions under different physiological and pathological conditions, with the open and closed states of the channel directly affecting mitochondrial energy metabolism efficiency and cell survival status.

The structural complexity of VDAC1 determines its functional diversity. In the open state, VDAC1 preferentially transports organic anions such as ATP, adenosine diphosphate (ADP), and phosphate groups, and this selective transport is crucial for maintaining the cellular energy balance. In the closed state, the channel primarily transports cations such as potassium, sodium, and calcium ions, and this switching mechanism provides cells with sophisticated ionic homeostasis regulatory capabilities. Victor V. Lemeshko’s computational model confirms that only a small fraction of outer membrane VDACs forming HK-VDAC1-ANT (adenine nucleotide translocator) contact sites generate sufficient mitochondrial outer membrane potential to regulate free VDAC permeability [47]. This efficient potential generation mechanism indicates that the HK-VDAC1 interaction represents a complex bioelectrochemical regulatory system rather than simple enzyme-carrier binding.

**Figure 2.**
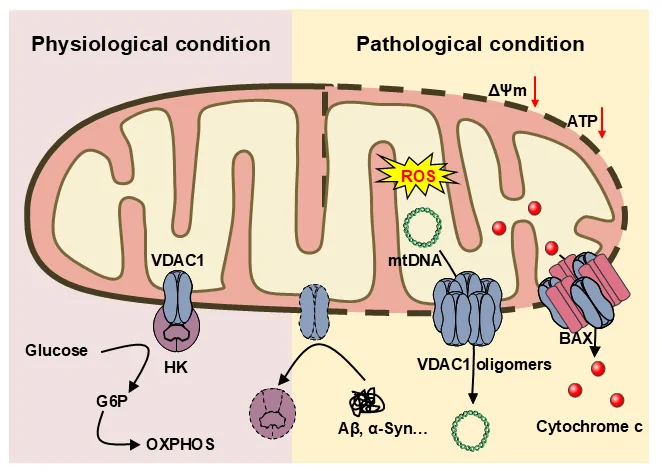
**HK-VDAC1 Metabolic Checkpoint: From Physiological Coupling to Pathological Disruption**. Physiological Condition: Under normal physiological conditions, HK forms a stable complex with VDAC1 at the mitochondrial outer membrane, creating a specialized metabolic checkpoint. This HK-VDAC1 interaction establishes a metabolic microdomain where glucose is efficiently phosphorylated to G6P using mitochondrial ATP, directly coupling glycolysis with OXPHOS. HK binding prevents VDAC1 oligomerization, maintaining mitochondrial membrane potential (ΔΨm) and membrane integrity, thereby blocking the release of pro-apoptotic factors such as cytochrome c and preserving neuronal viability. Pathological Condition: In neurodegenerative diseases, pathological proteins including Aβ, α-Syn, and other disease-associated proteins disrupt the HK-VDAC1 interaction through direct binding competition and conformational alterations. HK dissociation from VDAC1 triggers a cascade of mitochondrial dysfunction characterized by: (1) VDAC1 oligomerization and mitochondrial outer membrane permeabilization; (2) loss of mitochondrial membrane potential (ΔΨm) and impaired OXPHOS; (3) release of pro-apoptotic factors including cytochrome c and BAX; (4) mtDNA leakage into the cytoplasm; and (5) excessive ROS generation. This pathological cascade transforms the protective metabolic checkpoint into a death-signaling platform, establishing self-perpetuating cycles of bioenergetic collapse and neuronal degeneration that characterize the common pathophysiology of neurodegenerative diseases.

The interaction between HK and VDAC1 exhibits high specificity and regulatory control. Andrea Magrì et al. further discovered that the N-terminal peptide segment of HK1 (NHK1) is crucial for maintaining the functional state of VDAC1, not only ensuring efficient ATP utilization but also preventing the opening of the MPTP [48]. The precision of this interaction is manifested in the differential regulation of VDAC1 function by different structural domains of HK. The N-terminal peptide segment is primarily responsible for stable binding and channel regulation, whereas the catalytic domain influences the local metabolic microenvironment through its enzymatic activity, thereby modulating the functional state of VDAC1. Figure 2 illustrates the fundamental transition from physiological HK-VDAC1 coupling to pathological disruption that underlies mitochondrial dysfunction in neurodegenerative diseases. Under normal conditions, the stable HK-VDAC1 interaction maintains mitochondrial membrane integrity and efficient energy metabolism. However, pathological protein aggregates competitively displace hexokinase, triggering VDAC1 oligomerization and subsequent mitochondrial outer membrane permeabilization, which initiates the cascade of events leading to neuronal dysfunction and death.

The composition and regulatory mechanisms of the MPTP, a key regulatory point for cell death, have been a research hotspot. Raymond J. Winquist et al. conducted an in-depth analysis of the putative components of the MPTP, including VDAC, F1F0-ATP synthase, and its regulatory factor cyclophilin D (CyPD), and noted that these proteins provide new directions for drug development for various major diseases [49]. Notably, Olesoxime binding to VDAC1 has neuroprotective potential against NDs such as ALS, providing necessary experimental evidence for the development of therapeutic strategies based on HK-VDAC1 interactions [50, 51]. Vladimir M. Milenkovic et al. further refined our understanding of mitochondrial outer membrane protein complexes through their research on the 18 kDa translocator protein (TSPO) [52]. TSPO forms functional complexes with proteins, including VDAC, ANT, HK1/2, and glycogen synthase kinase-3 (GSK3β), which are essential components of the MPTP. The existence of such multiprotein complexes indicates that HK does not act on VDAC1 in isolation but instead participates in a more complex regulatory network [52]. Within this network, each protein component performs specific functions, whereas HK, which serves as a metabolic sensor, can regulate the activity of the entire complex according to the cellular energy status.

### 3.2 Regulatory Mechanisms of Mitochondrial Dynamic Homeostasis

In addition to structural interactions, HK participates in maintaining mitochondrial dynamic homeostasis through multiple regulatory mechanisms. HK participates in maintaining mitochondrial dynamic homeostasis through numerous mechanisms, and this regulation involves various processes, including mitochondrial fusion-fission, biogenesis, and quality control. Macarena S. Arrazola et al. demonstrated that Wnt signalling activation regulates mitochondrial HK2 localization and activity by inhibiting mitochondrial GSK-3β activity, preventing its dissociation from mitochondria [53]. The activation of this signalling pathway not only affects HK subcellular localization but also influences the overall functional state of mitochondria by modulating the intramitochondrial signal transduction network. When the glucose concentration is sufficient, the outer membrane potential generated by the HK1-VDAC1-ANT complex can drive calcium ion efflux from the intermembrane space, reduce the calcium ion concentration in the intermembrane space, inhibit MPTP opening, and increase neuronal survival rates [53].

The dynamic balance of mitochondrial fusion-fission is crucial for maintaining mitochondrial function, and HK plays a critical regulatory role in this process. Murali Vijayan et al. reported through gene regulatory experiments that a moderate reduction in VDAC1 expression could improve mitochondrial function in transgenic Tau mice [54]. In VDAC1 heterozygous knockout mice, the expression of the mitochondrial fusion genes Mitofusin-1 (Mfn1) and Mitofusin-2 (Mfn2) increased, the expression of the fission genes dynamin-related protein 1 (Drp1) and fission 1 (Fis1) decreased, HK expression significantly increased, and mitochondrial morphology and crista structure markedly improved. This phenomenon indicates that there is a complex regulatory relationship between HK expression levels and the balance of mitochondrial dynamics and that appropriate HK upregulation can promote the formation and maintenance of mitochondrial networks.

Mitochondrial biogenesis is another crucial aspect of maintaining mitochondrial function. Research by Maria Manczak et al. revealed that reducing VDAC1 expression could prevent mitochondrial dysfunction in AD models and that this protective effect was closely related to maintaining HK mitochondrial localization [55]. In VDAC1 heterozygous knockout mice, the expression of the mitochondrial fusion genes Mfn1 and Mfn2 is increased, the expression of the fission genes Drp1 and Fis1 is decreased, and HK expression is markedly upregulated [55]. Furthermore, these mice presented upregulated expression of mitochondrial biogenesis-related genes, including the mitochondrial transcription factor A (TFAM), nuclear respiratory factor 1 (NRF1), and peroxisome proliferator-activated receptor gamma coactivator 1-alpha (PGC-1α), suggesting that HK not only participates in maintaining mitochondrial function but also may regulate mitochondrial de novo synthesis [55].

The Wnt signalling pathway plays a crucial role in regulating mitochondrial function, and HK is an essential node in this regulatory network. Pedro Cisternas et al. further confirmed that Wnt signalling exerts neuroprotective effects in AD by activating glucose metabolism; enhancing the expression and activity of HK, phosphofructokinase (PFK), and AMP-activated protein kinase (AMPK); and promoting glycolysis and energy generation [56]. This protective mechanism involves not only direct regulation of metabolic enzyme activity but also indirect effects on mitochondrial biogenesis and quality control. Pedro Cisternas et al. reported that acute Wnt3a treatment could substantially increase neuronal glucose uptake, independent of changes in GLUT3 protein expression levels, but rather by increasing GLUT3 affinity and notably enhancing HK activity and the glycolytic rate [57].

The regulation of mitochondrial calcium homeostasis is another vital aspect of HK function. Under normal physiological conditions, mitochondria can buffer changes in cytoplasmic calcium ion concentrations, and this buffering capacity is highly dependent on the integrity and permeability of the mitochondrial outer membrane. Through its binding to VDAC1, HK not only regulates the transport of metabolites but also affects the transmembrane transport of calcium ions. When HK dissociates from VDAC1, the calcium buffering capacity of mitochondria decreases, easily leading to calcium overload and mitochondrial dysfunction [18, 28, 58]. This mechanism is fundamental in NDs, as neurons are susceptible to disruptions in calcium homeostasis.

### 3.3 Disease-Related Proteins and Mitochondrial Dysfunction

The HK-mitochondrial regulatory network becomes disrupted in neurodegenerative diseases through disease-specific pathological protein interactions. In different NDs, specific pathological proteins cause dysfunction by interfering with HK-mitochondrial interactions, and this interference mechanism exhibits distinct disease specificity. In AD, the interaction between Aβ and VDAC1 is a key mechanism leading to mitochondrial dysfunction. Angela Smilansky et al. confirmed that Aβ can directly bind to VDAC1, with preaggregated Aβ enhancing VDAC1 conductance and inducing its oligomerization, which in turn competitively displaces HK from its mitochondrial anchor, whereas nonaggregated Aβ reduces VDAC1 conductance [58]. This dual effect indicates that the Aβ aggregation state impacts on VDAC1 function: preaggregated Aβ disrupts the selective permeability of the mitochondrial outer membrane by promoting VDAC1 oligomerization to form large pores, whereas monomeric Aβ affects regular metabolite transport by blocking channels. A. Bobba et al. conducted in-depth studies and reported that Aβ1-42 exhibits noncompetitive inhibition of ANT-1, inducing mitochondria to produce large amounts of superoxide anions that oxidize ANT-1 active sites, leading to dysfunction [59]. This oxidative damage not only directly affects ANT-1 transport but also indirectly affects the function of other mitochondrial proteins by altering the redox state of the inner mitochondrial membrane. Notably, the tau peptide NH2-26-44 (NH2htau) competitively inhibits ANT-1, and when Aβ1-42 and NH2htau act together, the damage to ANT-1 is more severe, suggesting synergistic toxic effects between different pathological proteins. Bert C. Lynn et al. discovered through proteomic analysis that, from mild cognitive impairment to late-stage AD, the mitochondrial proteome shows progressive quantitative changes [27]. In particular, HK1 is considerably upregulated in the mild cognitive impairment (MCI) and early-onset Alzheimer's disease (EAD) stages, remains higher than that in controls, but slightly decreases in the late-onset Alzheimer's disease (LAD) stage, indicating an “HXH” expression pattern. VDAC1 also changes in an “HXH” pattern with disease progression, suggesting its essential role in energy metabolism and mitochondrial dysfunction. This temporal change pattern reflects the adaptive response of cells facing pathological stress, with early upregulation likely representing compensatory mechanisms, whereas later decline may reflect cellular functional exhaustion.

In PD, the interaction mechanism between α-syn and VDAC is more complex. Megha Rajendran et al. reported that the interaction between α-syn and VDAC occurs in two steps: first, the N-terminus binds to the mitochondrial membrane, and then, the C-terminus enters the VDAC pore under the action of voltage, leading to channel blockage [44]. This stepwise binding mechanism indicates that the impact of α-syn on VDAC function is a dynamic process; initial binding may not immediately affect channel function, but as protein conformation changes and further insertion occurs, channel function gradually becomes impaired. The N-terminal peptide of HK2 (HK2p) can competitively inhibit the binding of α-syn to the membrane, thereby blocking the complex formation between α-syn and VDAC and its transport process, providing essential clues for developing peptide-based therapeutic strategies. Ziba Dehghani et al. confirmed that α-syn fibrillization severs the crucial link between HK and mitochondria, whereas curcumin exerts protective effects by stabilizing HK-mitochondrial binding [25]. This protective mechanism involves not only direct protein-protein interactions but also the regulation of the intracellular redox status. Curcumin, a natural antioxidant, can reduce reactive oxygen species production and maintain the stability of the internal mitochondrial environment, thereby indirectly protecting the integrity of HK-VDAC1 interactions. Li Gao et al. demonstrated that Huangqin decoction can significantly increase brain cortical ATP content and mitochondrial complex I activity [60]. In the rotenone-induced PD model, the ketone body content in mitochondria was dramatically elevated, and glucose metabolism-related indicators decreased, whereas Huangqin decoction markedly reduced the ketone body content and the expression of its transporter MCT1; increased the levels of the glucose transporters GLUT1, citrate synthase, and HK; and activated the aerobic glycolysis pathway. This metabolic pattern switch indicates that traditional Chinese medicine formulas can rebalance mitochondrial energy metabolism through multitarget actions and restore normal HK function.

In ALS, mitochondrial dysfunction caused by SOD1 mutations has unique molecular mechanisms. Andrea Magrì et al. reported that SOD1-G93A can bind VDAC1 with high affinity and substantially reduce its channel activity [48]. The synthesized NHK1 peptide can specifically bind to VDAC1, efficiently block the binding between SOD1-G93A and VDAC1, restore the mitochondrial membrane potential, and notably improve the cell survival rate. This competitive binding mechanism provides a new perspective for understanding the pathogenesis of ALS and offers molecular targets for developing specific therapeutic drugs. Silvia Ravera et al. conducted studies on ALS patients and reported that synaptosomes from the spinal cord and motor cortex of SOD1-G93A mice presented impaired mitochondrial energy metabolism, which appeared in the early stages of the disease [13]. In synaptosomes, HK and phosphofructokinase activities were elevated, citrate synthase and malate dehydrogenase activities were increased, but lactate dehydrogenase activity did not significantly change. This selective change in enzyme activity indicates that metabolic reprogramming in ALS has apparent regional specificity and cell type specificity and that metabolic abnormalities in presynaptic regions may be early markers of motor neuron dysfunction.

The mitochondrial quality control mechanism in PD is also strongly affected by HK function. Research by David N. Hauser et al. on the HK-DJ-1-PINK1 (PTEN-induced putative kinase 1) pathway revealed a complex regulatory network of mitochondrial function [19]. DJ-1, a multifunctional protein, not only has antioxidant functions but also participates in mitochondrial quality control through its interaction with HK. María J. Contreras-Zárate et al. reported that PINK1 silencing inhibits insulin-like growth factor 1-mediated receptor activation and neuronal survival [61]. After PINK1 silencing, IGF-1R expression and its phosphorylation levels decreased, and the phosphorylation or expression of downstream signalling molecules, such as Akt, GSK3β, IRS-1, and HK, also substantially reduced, resulting in the formation of an interconnected signalling network. Naoki Tajiri et al. reported that cyclosporin A (CsA) can maintain mitochondrial integrity by increasing DJ-1 protein expression [62]. CsA blocks mitochondria-dependent cell death signals, prevents cytochrome C release from mitochondria, maintains mtDNA stability and the mitochondrial membrane potential, and prevents a decrease in ATP content [63]. This protective mechanism involves multiple levels of regulation, including transcriptional upregulation of DJ-1, increased protein-level stability, and functional-level mitochondrial protection. Huma Quadir et al. reported that Rho-associated protein kinase (ROCK) inhibitors can increase PINK1/Parkin-mediated mitophagy, promote HK2 recruitment to mitochondria, and increase Parkin localization and the engulfment and degradation of damaged mitochondria [64]. These findings reveal the close connection between cytoskeletal regulation and mitochondrial quality control. ROCK, as a key kinase in cytoskeletal regulation, affects not only cell morphology and movement through its active state but also the cellular energy metabolic status by regulating mitochondrial dynamics and HK localization.

HK dysfunction under metabolic disorder conditions also exacerbates mitochondrial damage. Pasquale Picone et al. revealed that metformin treatment leads to elevated intracellular reactive oxygen species levels, decreased mitochondrial membrane potential, and reduced HK2 and cytochrome C expression [65]. Through the activation of oxidative stress and nuclear factor kappa-light-chain-enhancer of activated B cells (NF-κB) signalling pathways, it increases amyloid precursor protein (APP) expression and processing, forming a vicious cycle. This drug-induced metabolic disorder model reveals the complex interactions between HK function and inflammatory signalling pathways, providing essential clues for understanding the comorbidity mechanisms between metabolic diseases and NDs. Moussa B. H. Youdim et al. demonstrated that rasagiline and its derivatives exert neuroprotective effects by delaying the opening of the mitochondrial VDAC, reducing MPTP opening, blocking PK11195 binding to proapoptotic peripheral benzodiazepine receptors, and acting synergistically with Bcl-2, HK, porin, and ANT on VDACs [66]. This multitarget protective mechanism indicates that effective neuroprotective strategies require simultaneous regulation of multiple mitochondrial function-related proteins, with HK serving as a key node whose functional state directly affects the efficacy of the entire protective network.

In conclusion, the HK-mediated mitochondrial regulatory network exhibits remarkable complexity and disease specificity in NDs. Through structural interactions with VDAC1, HK regulates mitochondrial energy metabolism while participating in homeostasis maintenance and quality control. Disease-specific pathological proteins disrupt this network through distinct mechanisms, leading to mitochondrial dysfunction and neuronal death. Understanding these mechanisms provides crucial insights into ND pathogenesis and offers a theoretical foundation for developing mitochondrion-targeted therapeutic strategies.

**Figure 3.**
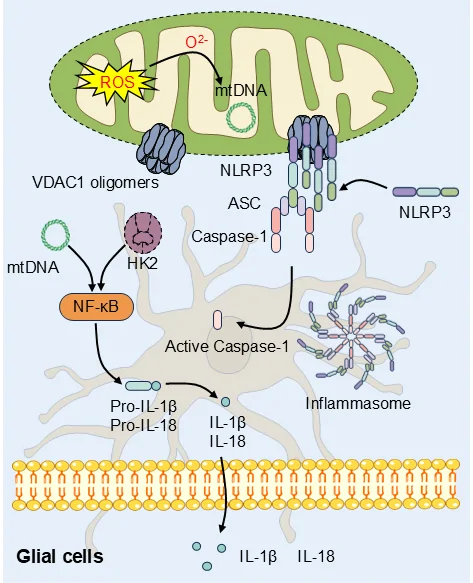
**Neuroinflammatory Cascade Following HK-VDAC1 Axis Disruption**. HK-VDAC1 axis disruption by pathological proteins triggers mitochondrial outer membrane permeabilization and VDAC1 oligomerization, resulting in the cytosolic leakage of DAMPs, including mtDNA, and a surge in O_2_^-^production. This ectopic mtDNA serves as an endogenous danger signal that activates NLRP3 inflammasomes in glial cells, while HK2 dissociation facilitates inflammasome assembly through NLRP3-ASC complex formation. Concurrent NF-κB activation drives pro-IL-1β and pro-IL-18 expressions. Assembled inflammasomes recruit and activate caspase-1, which processes pro-inflammatory precursors into mature IL-1β and IL-18. These cytokines establish a self-perpetuating neuroinflammatory microenvironment that amplifies glial activation, enhances oxidative stress, and promotes further HK-VDAC1 disruption, creating feed-forward cycles that transform acute metabolic dysfunction into chronic neurodegeneration across diverse neurodegenerative diseases.

## 4. The Role of HK in the Neuroinflammation-Oxidative Stress Axis

### 4.1 HK-Inflammasome Signalling Axis

HK serves as a critical molecular switch in inflammasome regulation through its interaction with VDAC1. Sung Hoon Baik et al. reported breakthrough findings demonstrating that the detachment of HK from its mitochondrial anchor promotes VDAC oligomerization, which triggers NOD-like receptor protein 3 (NLRP3) inflammasome assembly [20]. Superresolution microscopy revealed the complete activation cascade: various activators induce rapid HK2 dissociation from mitochondria, subsequently activate ER IP3 receptors, and trigger calcium release with subsequent mitochondrial uptake. Elevated mitochondrial matrix calcium drives VDAC oligomerization, leading to the formation of large pores that permit the egress of mtDNA from the mitochondrial matrix into the cytosol. Finally, VDAC oligomers synergistically recruit NLRP3 with this released-mtDNA to assemble active complexes. Figure 3 illustrates the complete neuroinflammatory cascade initiated by the HK-VDAC1 axis disruption. As demonstrated, the sequential events from HK dissociation to NLRP3 inflammasome activation involve multiple cellular compartments and signaling pathways. The release of mitochondrial DAMPs, particularly mtDNA, serves as a critical bridge connecting metabolic dysfunction to inflammatory activation in glial cells, ultimately leading to the production of mature inflammatory cytokines IL-1β and IL-18 that propagate neuroinflammation throughout the brain parenchyma.

This HK-mediated inflammatory activation mechanism exhibits high temporal specificity and disease-dependent variations. In AD, Aβ-induced HK dissociation activates inflammasomes through mitochondrial dysfunction, whereas in PD, α-syn fibrillar products trigger similar pathways with distinct kinetic profiles. Qun Yu et al. described dual regulatory mechanisms: direct disruption of HK-VDAC interactions induces inflammasome assembly, whereas elevated glucose-6-phosphate promotes HK release from mitochondria [67]. Under chronic inflammatory conditions, HK-mediated metabolic alterations form positive feedback loops in which NLRP3 activation upregulates the expression of key glycolytic enzymes, promoting metabolic reprogramming to sustain inflammatory responses.

The transcriptional amplification of inflammatory signals involves the coordinated action of multiple regulatory factors. Y Gao et al. discovered the TNFα-YAP-p65-HK2 signalling axis: activated macrophages secrete TNFα, which binds TNFR1 to activate the IKK complex [68]. IKKβ/ε activation leads to YAP phosphorylation and stabilization, promoting YAP nuclear translocation to form transcriptional complexes with TEAD and p65. This complex synergistically activates HK2 gene transcription, with enhanced glycolysis supporting sustained inflammatory responses. This discovery reveals that HK functions not only as a downstream effector of inflammatory responses but also as a key regulatory node for inflammatory signal amplification.

The complexity of this signalling network is further reflected in its cell type-specific manifestations. Different neural cell populations exhibit distinct sensitivities and response patterns to HK-mediated metabolic-inflammatory signals, contributing to selective neuronal vulnerability and region-specific pathological changes characteristic of different NDs. While inflammasome activation represents one critical arm of HK-mediated neuroinflammation, oxidative stress forms another interconnected component of this pathological axis.

### 4.2 Oxidative stress cascade reactions

Oxidative stress and HK dysfunction form a vicious cycle that drives ND progression through multiple interconnected mechanisms. This interaction involves direct reactive oxygen species (ROS)-mediated damage, oxidative stress effects on HK function and localization, and impairment of antioxidant defense systems resulting from HK dysfunction.

Leonardo M. Saraiva et al. confirmed that Aβ triggers neuronal HK1 dissociation from mitochondria, directly leading to mitochondrial dysfunction and a surge in mitochondrial ROS production [31]. Critically, 2-deoxyglucose, an alternative HK substrate, completely blocked Aβ-induced ROS production and significantly restored cell viability. This discovery reveals HK’s essential role in maintaining mitochondrial antioxidant capacity and provides critical clues for developing metabolic regulation-based antioxidant therapeutic strategies.

The complexity of oxidative stress cascades is reflected in their coordinated effects on multiple organelle functions. Pasquale Picone et al. reported that, under metabolic disorder conditions, metformin activates the AMPK-NF-κB signalling pathway, leading to increased APP expression and dramatically elevated extracellular Aβ secretion [65]. Oxidative stress further exacerbates APP expression and processing through NF-κB pathway activation, whereas insulin dose-dependently antagonizes these effects by activating the phosphatidylinositol 3-kinase/protein kinase B (PI3K/AKT) pathway to reduce mitochondrial ROS levels significantly. These findings indicate that oxidative stress functions not only as a disease consequence but also as an essential driving factor for disease progression.

Disease-specific oxidative stress patterns reflect distinct pathological mechanisms. Lyudmila A. Tikhonova et al. investigated the effects of Aβ25-35 on red blood cells and reported that oxidative stress disrupts membrane stability and antioxidant enzyme systems, leading to energy metabolism disorders [69]. In aged red blood cells, glutathione peroxidase and glutathione transferase activities are considerably diminished, increasing their sensitivity to Aβ25-35 toxicity. This age-related increase in oxidative stress susceptibility may explain why NDs primarily affect elderly populations. P. Hemachandra Reddy reported that Aβ activates GSK3β, leading to VDAC1 phosphorylation, which interferes with HK mitochondrial localization and results in synaptic dysfunction and neuronal damage [70]. As a convergence point of multiple signalling pathways, GSK3β activation not only directly affects HK functional status but also amplifies oxidative stress damage by regulating the phosphorylation of other key proteins.

In PD, dopaminergic neurons exhibit special susceptibilities to oxidative stress related to their unique metabolic characteristics and HK functional status. G. Gille et al. reported that rotenone caused massive death of tyrosine hydroxylase-positive neurons by inhibiting mitochondrial complex I activity and increasing ROS production [42]. Ziba Dehghani et al. reported that α-syn fibrillar products induce HK detachment from mitochondria, accompanied by a marked elevation in mitochondrial ROS levels. Curcumin exerts protective effects by maintaining HK mitochondrial localization, significantly inhibiting α-syn-induced HK detachment and the subsequent rise in ROS [25]. This multilayered protective mechanism may explain why natural antioxidants demonstrate neuroprotective effects.

TCM formulations demonstrate comprehensive antioxidant effects through multitarget regulatory mechanisms. Studies have shown that Huangqin decoction significantly ameliorates rotenone-induced mitochondrial dysfunction, with substantial recovery of ATP content and dramatic enhancement of HK activity [60]. Importantly, this study identified ketone body metabolism abnormalities as novel PD pathological markers. In the model groups, acetoacetate and β-hydroxybutyrate levels were dramatically elevated, while these indicators significantly decreased after treatment, with increased GLUT1 expression and downregulated MCT1 expression, indicating the conversion from ketone body metabolism to normal glucose metabolism.

Research has demonstrated that CsA upregulates DJ-1 protein expression, maintaining mitochondrial integrity and reducing ischemia-induced oxidative stress damage [62]. CsA significantly enhances GSH activity, keeps the mitochondrial membrane potential, prevents cytochrome c release, and promotes DJ-1 translocation to mitochondria, increasing the local antioxidant capacity [63]. These findings emphasize the vital role of mitochondrial quality control systems in antioxidant defense.

In ALS, oxidative stress caused by SOD1 mutations and HK functional abnormalities results in the formation of a unique pathological circuit. Andrea Magrì et al. reported that NHK1 can rescue mitochondrial dysfunction caused by SOD1 G93A [14]. High-resolution respirometry revealed that SOD1 G93A caused a severe decrease in ATP-linked respiration, which was substantially restored after NHK1 treatment. Moreover, mitochondrial SOD1 accumulation was markedly reduced, revealing HK’s important role in maintaining normal mitochondrial function and providing insights into motor neuron vulnerability.

### 4.3 Glial Cell Metabolic-Inflammatory Coupling

Glial cells exhibit distinct metabolic reprogramming patterns that determine their inflammatory phenotypes and functional contributions to neurodegeneration. Unlike the general inflammasome mechanisms described above, this section focuses on cell-type-specific metabolic-inflammatory coupling mechanisms.

#### 4.3.1 Microglial Polarization Control

Metabolic reprogramming drives microglial activation states through specific temporal and functional patterns. Junjie Cheng et al. confirmed through temporal analysis that early glycolytic reprogramming is a key event in microglial inflammatory activation [71]. Glycolysis markedly increased within hours after lipopolysaccharide (LPS) stimulation, with increased expression of HK2 and GLUT1. This study elucidated the complete glycolysis-mTOR-NF-κB signalling axis: 2-DG significantly inhibited LPS-induced mTOR phosphorylation, prevented NF-κB p65 nuclear translocation, and simultaneously inhibited p65 acetylation through SIRT1 regulation.

Juan F. Codocedo et al. revealed the quantitative relationship between HK2 expression levels and microglial activation through gene dosage studies [30]. The HK2 heterozygous deletion had protective effects: it significantly reduced dense-core plaques, decreased Iba1+ cell coverage, reduced TREM2 levels, and shifted microglial gene expression toward a homeostatic phenotype. However, complete HK2 deletion had detrimental effects: increased plaque numbers and exacerbated inflammation. Transcriptomic analysis revealed that HK2 heterozygous deletion was associated with increased IκBα levels, which inhibited NF-κB nuclear translocation and inflammasome gene expression. This dose-dependent effect indicates an optimal HK2 expression range for the regulation of microglial function.

Mengqi Fang et al. explored disease-specific manifestations of glucose metabolic reprogramming in microglia [16]. In AD, increased glycolysis is characterized by increased HK and PFK activity, whereas impaired OXPHOS manifests as decreased citrate synthase levels. TREM2 mutations lead to reduced glucose metabolism and cerebral blood flow. In PD, increased glycolysis is characterized by increased expression of HK2, LDH, and pyruvate kinase M2 (PKM2), with α-syn activating PKM2 to promote glycolysis and inhibit OXPHOS through the AKT-mTOR-HIF-1α pathway.

Xin Zhong et al. reported that cordycepin achieves anti-inflammatory effects through dual targeting of HK2 and pyruvate dehydrogenase kinase 2 (PDK2) [72]. Cordycepin selectively distributes to microglial mitochondria, binds explicitly to HK2 and PDK2, promotes both glycolysis and oxidative phosphorylation, and induces the microglial transition from the M1 phenotype to the M2 phenotype. This dual metabolic enhancement provides sufficient energy support for maintaining the M2-type anti-inflammatory phenotype.

#### 4.3.2 Astrocytic metabolic support

Astrocytes play crucial roles in neuroinflammation through their metabolic support functions and inflammatory responses. Ik Dong Yoo et al. revealed that elevated expression of CLOCK and BMAL1 in AD leads to impaired aerobic glycolysis in astrocytes, with substantially decreased protein levels of HK1 and lactate dehydrogenase A, affecting the energy supply of neurons [73]. The influence of circadian rhythm regulatory proteins on metabolic enzyme expression suggests that circadian rhythm disruption may contribute to ND pathogenesis by impacting glial cell metabolic functions. Shuangxue Han et al. confirmed that CY-09 significantly improves brain glucose metabolic function by inhibiting NLRP3 inflammasome activation and restoring HK1 and HK2 expression and mitochondrial binding [74]. Through 18F-FDG PET imaging and metabolomics analysis, inflammation suppression led to significant improvements in brain glucose utilization: the whole-brain SUV was significantly restored, glucose uptake in the cortical and hippocampal regions was markedly improved, and the brain glucose metabolic rate was increased considerably.

#### 4.3.3 Oligodendrocytic Glycolytic Stress

Oligodendrocytes exhibit unique vulnerability to metabolic stress due to their high-energy-demanding myelin synthesis and maintenance functions [26, 75]. Xinwen Zhang et al. discovered that glycolytic stress in oligodendrocytes triggers inflammasome activation through a disease-specific pathological circuit [75]. This study revealed a complete Drp1-HK1-NLRP3 signalling axis: Aβ stimulation leads to excessive Drp1 activation, increased mitochondrial fission inhibits HK1 activity, and glycolytic defects trigger metabolic stress and ultimately activate the NLRP3 inflammasome [26].

In 5XFAD mice, HK activity is reduced in mature oligodendrocytes, while methylene blue treatment can significantly improve HK activity and glucose uptake in astrocytes [75]. As the primary cell type responsible for myelin formation and maintenance, oligodendrocyte metabolic dysfunction not only affects neural conduction but also influences surrounding neurons and other glial cells through inflammatory factor release [75].

#### 4.3.4 Glial-neuronal communication networks

The interaction between glial cell activation and neuroinflammation involves multiple types of intercellular signalling communication. Damage-associated molecular patterns (DAMPs) released by neurons activate microglia and astrocytes, whereas inflammatory factors released by activated glial cells further damage neurons, forming vicious cycles [76]. HK not only regulates the metabolic state of glial cells themselves but also influences intercellular signalling communication by modulating inflammatory factor production. This complex interaction network emphasizes that treating NDs requires the coordinated regulation of multiple cell types rather than targeting single cell types or molecular targets. The cell type-specific metabolic-inflammatory coupling mechanisms provide critical theoretical foundations for the development of comprehensive therapeutic strategies targeting glial cell dysfunction in neurodegeneration.

In conclusion, HKs orchestrate multilevel, multidimensional regulatory networks within the neuroinflammation-oxidative stress axis. Through regulating inflammasome assembly and activation, HK participates in the initiation and amplification of the inflammatory response. By maintaining mitochondrial function and antioxidant defense, HK regulates oxidative stress generation and clearance. Through controlling metabolic reprogramming and the inflammatory phenotypes of glial cells, HK influences neuroinflammation persistence and resolution. In different NDs, these mechanisms exhibit disease-specific characteristics, providing necessary theoretical foundations for developing targeted therapeutic strategies.

## 5. Molecular Mechanisms of HK Regulation in Neuronal Fate Determination

### 5.1 Cell death signal transduction networks

HK functions as a critical molecular switch in neuronal fate determination, integrating multiple signalling pathways that govern cell survival and death decisions. In addition to its enzymatic role in glucose metabolism, HK serves as a key regulatory node where metabolic status directly influences cellular survival outcomes through sophisticated feedback mechanisms. The precision of HK-mediated cell fate control is exemplified by glucose-6-phosphate feedback regulation. A. Bobba et al. reported that glucose-6-phosphate produced by HK can directly regulate the apoptotic balance in cerebellar granule cells [77]. When glucose-6-phosphate accumulation reaches threshold concentrations, it disrupts the protective binding between HK1 and VDAC1, leading to MPTP opening and triggering apoptotic cascades. This mechanism reveals that HK enzymatic products function as signalling molecules, creating a sophisticated feedback loop where metabolic flux directly determines cell fate. When metabolic product accumulation exceeds cellular processing capacity, it reciprocally affects HK’s own functional state, establishing a critical metabolic checkpoint for neuronal survival decisions.

The PI3K/AKT survival pathway represents another crucial node where HK integrates survival signals with metabolic competence. Gonzalo Arboleda et al. demonstrated that ceramide-induced neuronal death involves early inhibition of PI3K/AKT signalling, which directly affects HK activity regulation [78]. Ceramide inhibits PI3K activity, reducing AKT phosphorylation and compromising HK enzymatic function, resulting in decreased glycolytic efficiency and insufficient mitochondrial substrate supply. This cascade illustrates how external death signals are amplified through metabolic pathways, with HK serving as both a target and amplifier of survival/death signals. The PI3K/AKT-HK axis thus creates a multilayered regulatory network in which survival signals must maintain both direct cellular protection and metabolic homeostasis.

Stress-induced compensatory responses reveal the adaptive nature of HK-mediated fate determination in neuronal systems. A. Bobba et al. reported that during early apoptotic stages in cerebellar granule cells, the upregulation of glycolytic enzyme activity occurs synchronously with alterations in mitochondrial function, including substantial increases in HK activity [17]. This compensatory upregulation represents a protective mechanism for neurons in response to an energy crisis and is correlated with autophagy pathway activation. However, when stress exceeds the adaptive capacity of neurons, this compensatory mechanism fails, leading to irreversible cell death. This dual nature of HK responses highlights the delicate balance between adaptive survival mechanisms and pathological dysfunction in neuronal tissue.

**Figure 4.**
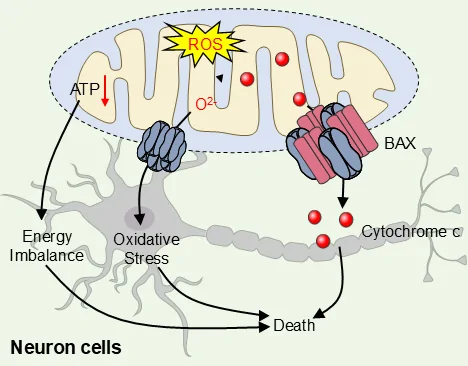
**Mitochondrial Permeabilization and Neuronal Death Cascade**. HK dissociation from VDAC1 triggers mitochondrial outer membrane permeabilization through VDAC1 oligomerization, initiating a coordinated apoptotic cascade in neuronal cells. Membrane permeabilization facilitates the cytosolic release of pro-apoptotic factors including cytochrome c and BAX, while simultaneously compromising mitochondrial ATP synthesis capacity, resulting in severe energy imbalance. The released cytochrome c activates caspase-dependent apoptotic pathways, while impaired bioenergetics triggers compensatory mechanisms that paradoxically amplify the burden of oxidative stress (O_2_^-^and ROS). This escalating cycle of oxidative damage creates a self-perpetuating cycle where ROS-induced mitochondrial damage further compromises ATP generation and promotes additional pro-apoptotic factor release. The convergence of energy depletion and oxidative stress establishes an irreversible commitment to neuronal death, representing the final common pathway through which diverse neurodegenerative insults ultimately converge to eliminate vulnerable neuronal populations.

Inflammatory signalling networks represent an additional critical dimension of HK-mediated fate control in neurodegeneration. Accumulating evidence from studies on NLRP3 inflammasome activation reveals that HK dissociation from mitochondria promotes VDAC oligomerization, facilitating inflammasome assembly and activation [20]. This process creates feedback loops between metabolic dysfunction and inflammatory responses, where compromised HK function renders neurons more susceptible to inflammatory damage, while inflammatory mediators can further impair HK activity. These inflammatory-metabolic interactions establish a vicious cycle that accelerates neuronal death, underscoring HK’s importance in maintaining neuronal resilience against multiple pathological stressors. Figure 4 illustrates the convergence of numerous death signaling pathways that culminate in neuronal apoptosis following HK-VDAC1 axis disruption. As demonstrated, the simultaneous occurrence of bioenergetic failure, oxidative stress amplification, and mitochondrial outer membrane permeabilization creates an irreversible commitment to cell death. The release of pro-apoptotic factors, including cytochrome c and BAX, combined with severe energy imbalance and ROS accumulation, drives neurons beyond the point of metabolic rescue, representing the final common pathway through which diverse neurodegenerative insults ultimately converge to eliminate vulnerable neuronal populations.

### 5.2 Mitochondrial-Nuclear Signalling Axis

While cell death signal transduction networks demonstrate HK’s role in immediate fate decisions, the mitochondrial-nuclear signalling axis reveals how HK coordinates long-term cellular adaptation strategies. HK occupies a central position in the mitochondrial-nuclear communication network, serving as both a sensor of mitochondrial functional status and a regulator of nuclear gene expression programs that determine cellular fate. This bidirectional signalling system enables neurons to coordinate mitochondrial bioenergetics with nuclear transcriptional responses, creating an integrated cellular response to metabolic challenges [18, 20].

Mitochondrial quality control signalling pathways exemplify HK’s role in mitochondrial-nuclear communication. David N. Hauser et al. revealed that HK1 redistribution between the mitochondrial and cytoplasmic compartments serves as a critical signal for mitochondrial dysfunction [19]. Under DJ-1-deficient conditions, HK1 dissociation from the outer mitochondrial membrane triggers metabolomic changes, including elevated sorbitol and fructose levels, signalling that the nuclear transcriptional machinery initiates compensatory responses. This redistribution process is age dependent, suggesting that HK-mediated signalling becomes increasingly critical for maintaining mitochondrial-nuclear communication during neuronal aging.

Mitochondrial biogenesis represents a key nuclear response regulated by HK-mediated signals. Findings from PINK1 studies demonstrate that PINK1 silencing inhibits insulin-like growth factor 1-mediated receptor activation, affecting the expression of downstream signalling molecules, including HK [61]. These findings reveal that mitochondrial quality control proteins not only participate in damaged mitochondrial clearance but also regulate mitochondrial biogenesis through growth factor signalling pathways. When these quality control mechanisms are compromised, neurons lose their ability to maintain mitochondrial homeostasis, with HK serving as both a target and a mediator of mitochondrial biogenesis signals.

The precision of mitochondrial-nuclear signalling is reflected in the dynamic regulation of mitochondrial networks. Murali Vijayan et al. demonstrated that a reduction in VDAC1 enhances the expression of mitochondrial fusion genes (Mfn1 and Mfn2) while decreasing the expression of fission genes (Drp1 and Fis1), accompanied by significant HK upregulation [54]. This finding indicates that mitochondrial channel expression levels require precise calibration, with HK serving as both a sensor of this balance and an effector of compensatory responses. The HK-VDAC1 interaction thus functions as a critical node integrating mitochondrial dynamics with metabolic competence in neuronal systems.

Mitochondrial genome stability represents another crucial aspect of the mitochondrial-nuclear communication mediated by HK. Studies have demonstrated that CsA treatment maintains mtDNA stability through DJ-1 upregulation and that HK function is stabilized in neuronal systems [62]. Since mtDNA encodes essential respiratory chain components, its stability is fundamental for neuronal mitochondrial function. HK dissociation from mitochondria not only affects energy metabolism but also may influence mtDNA stability by altering the mitochondrial microenvironment, creating a feedback loop in which metabolic dysfunction amplifies genetic instability in neurons.

Disease-specific alterations in mitochondrial-nuclear signalling reveal the pathological significance of this communication network in neurodegeneration. Traditional Chinese medicine interventions can restore the mitochondrial-nuclear signalling balance in PD models, reducing ketone body content while increasing glucose metabolism indicators, including HK levels [60]. This therapeutic restoration of metabolic balance demonstrates that mitochondrial-nuclear communication mediated by HK represents a viable therapeutic target for treating neurodegeneration, with implications for the development of precision medicine approaches.

### 5.3 Temporal dynamics and cellular adaptation mechanisms

The regulation of neuronal fate by HKs involves sophisticated temporal control mechanisms that integrate cellular stress responses, adaptive programming, and disease progression dynamics. This temporal regulation ensures that neuronal fate decisions evolve appropriately in response to changing environmental challenges and pathological conditions throughout the lifespan.

The temporal coordination of cellular quality control systems represents a critical aspect of HK-mediated fate determination in neurons. During cellular stress responses, HK function undergoes dynamic changes that coordinate with autophagy activation and mitochondrial quality control mechanisms. Murali Vijayan et al. demonstrated that a reduction in VDAC1 enhances autophagy activity and significantly increases PINK1, Parkin, and the LC3B-II/LC3B-I ratio, accompanied by HK upregulation [54]. The temporal sequence of these responses determines whether neurons successfully adapt to stress or progress toward death, with early HK dysfunction potentially triggering protective autophagy responses, whereas prolonged dysfunction leads to autophagy failure and cellular death.

The autophagy-lysosomal system functions as a key temporal executor of HK-mediated fate decisions in neuronal systems. Simon M. Bell et al. demonstrated that HK1 overexpression in sporadic AD astrocytes stabilizes calcium homeostasis and improves mitochondrial network integrity, with these benefits mediated through enhanced autophagy function [29]. HK1 overexpression not only directly improves energy metabolism but also promotes long-term cellular maintenance programs essential for neuronal survival. These immediate and long-term effects of HK regulation underscore the temporal complexity of neuronal fate determination. These findings reveal that HK influences neuronal fate through both immediate metabolic effects and sustained adaptive responses that evolve over extended time periods.

Age-related evolution of HK-mediated fate control reveals the changing vulnerability landscape of neurons across the lifespan. The age-dependent characteristics of HK regulatory mechanisms suggest that neuronal fate determination systems become increasingly fragile during aging, requiring more precise regulation to maintain cellular viability. This temporal evolution of vulnerability may explain why neurodegenerative diseases typically manifest during aging, when the cellular energy demands exceed the capacity of increasingly dysfunctional HK-mediated metabolic systems, and why early-life metabolic programming can influence late-life neurodegeneration risk.

Disease progression dynamics further illustrate the temporal aspects of HK-mediated fate control in neurodegeneration. In different neurodegenerative conditions, the timing and sequence of HK dysfunction are correlated with disease severity and progression rates [24, 25, 35, 40]. Early disease stages may be characterized by compensatory HK upregulation as neurons attempt to maintain energy homeostasis, whereas advanced stages are characterized by progressive HK dysfunction and loss of adaptive capacity. This temporal progression pattern suggests that the therapeutic window for HK-targeted interventions may be most effective during early disease stages when compensatory mechanisms remain functional.

Activity-dependent metabolic coupling represents another crucial temporal aspect of HK-mediated fate control in neurons. Synaptic transmission efficiency and long-term potentiation are closely linked to HK function and mitochondrial energy production, creating a dynamic system in which neuronal fate is continuously influenced by functional activity levels [17]. The temporal coordination between synaptic activity, energy demand, and HK regulation establishes activity-dependent survival mechanisms [56, 57]. Disruption of this activity-metabolism coupling may represent an early pathological event in neurodegeneration that precedes overt cell death, suggesting that maintaining neuronal activity could be neuroprotective through HK-mediated mechanisms [17, 56, 57].

The molecular mechanisms by which HK regulates neuronal fate determination reveal a sophisticated multilayered control system that integrates metabolic status, organellar function, and cellular quality control through signal transduction networks, mitochondrial-nuclear communication axes, and temporal coordination systems. Understanding these mechanisms provides crucial insights into the pathogenesis of neurodegenerative diseases and establishes HK as a central regulator of neuronal survival and death decisions, with significant implications for developing precision therapeutic strategies that can modulate neuronal fate at multiple regulatory levels through temporal and mechanistic targeting approaches.

## 6. Therapeutic Strategies Targeting HK in Neurodegeneration

Based on a comprehensive understanding of the central role of HK in neurodegeneration, therapeutic strategies targeting HK have emerged as promising interventions for neurodegenerative diseases. These approaches can be systematically categorized into five complementary strategies: direct HK-targeting therapeutics, indirect HK modulation strategies, integrated combination therapies, precision medicine approaches, and advanced delivery technologies, each offering distinct mechanisms and therapeutic advantages.

### 6.1 Direct HK-Targeting Therapeutic Approaches

Direct therapeutic interventions achieve neuroprotection through precise modulation of HK enzymatic activity, stabilization of its mitochondrial localization, or functional mimicry of its protective properties [30, 32]. These approaches represent the most mechanistically targeted strategies for HK-based neurotherapeutics (Table.1).

**Table 1:** Therapeutic Strategies Targeting HK in Neurodegeneration: Mechanisms and Applications.

Therapeutic strategy	Category	Therapeutic Agent	Mechanism of Action	Primary Target	Disease Models	Key Advantages	Ref
Direct HK Targeting	*Peptide Therapeutics*	N-terminal hexokinase 1 peptide (NHK1)	VDAC1 binding SOD1 G93A-VDAC1 disruption	VDAC1	ALS models	Greater efficiency than full-length HK1 Restores mitochondrial membrane potential Enhances cell viability	[14, 48]
Direct HK Targeting	*Peptide Therapeutics*	HK2-derived peptide (HK2p)	Competitive inhibition of α-synuclein membrane binding	α-synuclein-VDAC interaction	Parkinson's disease models	Dose-dependent reduction of α-synuclein-induced VDAC blockade Restoration of VDAC open states	[25]
Direct HK Targeting	*Small Molecules*	2-Deoxyglucose (2-DOG)	Glucose analog Alternative HK substrate	HK enzymatic activity	AD models	Completely abolishes amyloid-β-induced ROS generation Maintains glycolytic flux Suppresses LPS-induced mTOR phosphorylation	[31, 71, 79]
Direct HK Targeting	*Gene Therapy*	HK1 overexpression	Gene overexpression	HK1 expression	Sporadic Alzheimer's disease	Ameliorates astrocytic mitochondrial function Restored total ATP levels Increased extracellular lactate secretion	[29]
Direct HK Targeting	*Gene Therapy*	HK2 gene transfer	Gene overexpression	HK2 expression	Rotenone and MPTP mouse models of PD	Prevents neurodegeneration	[22]
Indirect Modulation	*Signaling Pathways*	Andrographolide	Activates Wnt Regulates mitochondrial hexokinase II localization Inhibits mitochondrial GSK-3β	HK and phosphofructokinase activities	AD models	Prevents HK dissociation from mitochondria Restores hexokinase activities	[56]
Indirect Modulation	*Signaling Pathways*	Lithium carbonate	Activates Wnt Regulates mitochondrial hexokinase II localization Inhibits mitochondrial GSK-3β	HK and phosphofructokinase activities	AD models	Prevents HK dissociation from mitochondria Restores hexokinase activities	[56]
Indirect Modulation	*Signaling Pathways*	Cyclosporine A	DJ-1 protein upregulation Mitochondrial protection	Mitochondrial integrity	Ischemic models	Maintains mitochondrial integrity Blocks mitochondria-dependent cell death Prevents cytochrome c release	[62]
Indirect Modulation	*Natural Compounds*	Huangqin decoction	Multi-target metabolic regulation	ATP synthesis, mitochondrial complex I	Parkinson's disease models	Elevates cerebral cortical ATP content Enhances glucose transporter GLUT1 Enhances hexokinase levels	[60]
Indirect Modulation		Cordycepin	Targeting HKII and PDK2	HKII and PDK2	Microglial models	Selective distribution to microglial mitochondria Promotes glycolysis and oxidative phosphorylation Induces M1 to M2 polarization	[72]
Indirect Modulation		Hydroxycinnamic acid components	Gene expression upregulation	Hexokinase 1 and phosphofructokinase M	Neurotoxicity models	Upregulate the hexokinase 1 gene expression Enhanced neuronal cell viability	[80]
Combination Strategies	*Inflammation-Metabolism*	CY-09	NLRP3 inflammasome inhibition	NLRP3 inflammasome	3×Tg-AD mice	Improves cerebral glucose metabolic function Restoration of HK1 and HK2 expression Marked improvement in glucose utilization rates	[74]
Combination Strategies	*Mitochondrial Quality*	ROCK inhibitors	PINK1/Parkin-mediated mitophagy enhancement	ROCK pathway	Parkinson's disease	Promotes hexokinase 2 recruitment to mitochondria Enhances Parkin localization	[64]
Combination Strategies	*Growth Factor Integration*	Insulin	PI3K/AKT pathway activation	Insulin signaling pathway	Metabolic disorder models	Inhibits NF-κB activation Reduces APP expression Decreases mitochondrial ROS levels	[65]
Precision Medicine	*Personalized Approaches*	Precise HK expression modulation	Dose-dependent HK2 expression control	HK2 expression levels	Microglial activation models	HK2 heterozygous deficiency confers protective effects Avoids complete deficiency detrimental outcomes	[30]
Advanced Delivery	*Mitochondrial Targeting*	Triphenylphosphonium (TPP)-conjugated carriers	Mitochondria-targeted delivery	Mitochondrial compartments	Multiple models	10-100 fold enrichment in mitochondrial compartments Enhanced therapeutic potency Minimized off-target effects	[82, 83]
Advanced Delivery	*Mitochondrial Targeting*	Mitochondria-penetrating peptides (MPPs)	Cell-penetrating sequences with mitochondrial targeting	Mitochondrial matrices	Multiple applications	Delivery of larger therapeutic payloads Precise subcellular localization	[84, 85]
Advanced Delivery	*BBB Penetration*	Focused ultrasound-mediated delivery	Transient, reversible blood-brain barrier opening	Blood-brain barrier	CNS delivery	Enhanced CNS penetration Minimal adverse effects	[86, 87, 88]
Advanced Delivery	*BBB Penetration*	Receptor-mediated transcytosis	Endogenous transport mechanisms	Transferrin and insulin receptors	CNS delivery	Achieve therapeutic CNS concentrations Maintaining systemic safety profiles	[89, 90, 91]
Advanced Delivery	*Controlled Release*	PLGA-based systems	Sustained release	Long-term delivery	Chronic conditions	Sustained release over weeks to months Reducing dosing frequency Maintaining consistent therapeutic levels	[92, 93, 94]
Advanced Delivery	*Controlled Release*	Implantable delivery devices	Programmable release profiles	CNS drug delivery	Long-term CNS delivery	Programmed for specific release profiles Refillable as needed Minimizing systemic exposure	[95, 96]

#### 6.1.1 Molecular peptide therapeutics

The development of HK-derived peptides has provided compelling proof-of-concept validation for direct HK-targeting strategies. NHK1 exemplifies this approach through its specific binding affinity for VDAC1 and subsequent modulation of channel activity. Research has demonstrated that the NHK1 peptide is approximately 20-fold more efficient than full-length HK1 in disrupting pathological SOD1 G93A-VDAC1 interactions [48]. In the ALS model, NHK1 treatment successfully restored the mitochondrial membrane potential, ameliorated mitochondrial dysfunction, and significantly increased cell viability. High-resolution respirometry analysis revealed substantial recovery of ATP-linked respiratory function, demonstrating the peptide’s capacity to restore bioenergetic homeostasis [14].

Similarly, HK2p competitively inhibits α-syn membrane binding in PD models [25]. This peptide dose-dependently reduces α-syn-induced VDAC blockade events and restores the open state of VDAC, effectively preventing α-syn-mediated mitochondrial dysfunction [25]. The therapeutic advantages of these peptide-based agents include high specificity, minimal off-target effects, and precise targeting of the HK-VDAC interaction interface, positioning them as valuable tools for precision medicine approaches in treating neurodegeneration.

#### 6.1.2 Enzymatic activity modulators

Small molecule modulators of HK activity represent another promising direct targeting approach. 2-DG, which functions as a glucose analogue and alternative HK substrate, has remarkable neuroprotective potential through multiple mechanisms [31]. Research has revealed that 2-DG completely abolishes Aβ-induced reactive oxygen species generation, resulting in significant restoration of neuronal viability [79]. The protective mechanism involves the capacity of 2-DG to maintain glycolytic flux while preventing pathological HK dissociation from mitochondria. In addition to exerting direct metabolic effects, 2-DG exerts dual anti-inflammatory and neuroprotective effects by modulating microglial activation states [71]. Through glycolysis inhibition, 2-DG significantly suppresses LPS-induced mTOR phosphorylation and subsequently inhibits NF-κB signalling pathway activation, demonstrating the connection between metabolic modulation and neuroinflammation control [71].

#### 6.1.3 Gene therapy strategies

Gene therapy approaches employing HK overexpression have demonstrated promising therapeutic efficacy across multiple neurodegenerative models. In sporadic AD studies [29], increased HK1 expression significantly ameliorated astrocytic mitochondrial function and glycolytic activity. This intervention leads to restored total ATP levels, increased extracellular lactate secretion for neuronal support, and improved mitochondrial membrane potential [29]. Similarly, HK2 gene transfer prevents neurodegeneration in both rotenone and MPTP mouse models of PD, demonstrating broad applicability across different pathological contexts [22]. These findings collectively underscore the therapeutic versatility of direct HK-targeting approaches, ranging from small-molecule modulators to sophisticated gene delivery systems, each offering unique advantages for specific clinical applications.

### 6.2 Indirect HK Modulation Strategies

While direct targeting approaches offer precision and specificity, indirect modulation strategies provide broader therapeutic coverage by addressing multiple pathological pathways simultaneously. Indirect therapeutic approaches influence HK functional states through upstream signalling pathway regulation, cellular microenvironment optimization, or the restoration of metabolic homeostasis. These strategies offer the advantage of targeting multiple pathological processes simultaneously while maintaining the physiological regulation of HK activity.

#### 6.2.1 Signalling Pathway Modulation

Wnt signalling pathway activation represents one of the most promising indirect modulation approaches [56]. Research has demonstrated that Wnt3a regulates mitochondrial HK2 localization and activity by inhibiting mitochondrial GSK-3β activity, thereby preventing HK dissociation from mitochondria [56]. In AD models, treatment with the Wnt activator andrographolide and lithium carbonate restores HK and phosphofructokinase activities to wild-type levels while significantly enhancing neuronal glucose uptake capacity [56]. This protective effect operates independently of alterations in glucose transporter expression, instead achieving therapeutic benefits through increased GLUT3 affinity and HK activity [57]. The ability of the Wnt pathway to coordinate metabolic and survival signalling makes it an attractive target for comprehensive neuroprotective strategies. CsA exemplifies another essential class of indirect modulation strategies through mitochondrial protection mechanisms [62]. This compound maintains mitochondrial integrity through DJ-1 protein upregulation, blocking mitochondria-dependent cell death signalling, preventing cytochrome c release, and preserving the mitochondrial membrane potential and ATP content [62]. These effects indirectly support HK function by maintaining optimal mitochondrial microenvironments.

#### 6.2.2 Traditional Medicine and Natural Compounds

Traditional Chinese medicine formulations demonstrate unique advantages in indirect HK functional modulation through multitarget mechanisms [60]. Huangqin decoction significantly elevates cerebral cortical ATP content and mitochondrial complex I activity in PD models while simultaneously regulating ketone body metabolic abnormalities through reduced ketone body levels and decreased MCT1 transporter expression [60]. This formulation enhances the levels of the glucose transporters GLUT1, citrate synthase, and HK, thereby activating aerobic glycolytic pathways and restoring metabolic homeostasis [60].

Cordycepin achieves anti-inflammatory effects through dual targeting of HK2 and PDK2 [72], exhibiting selective distribution to microglial mitochondria with specific binding affinity for both targets. This compound promotes glycolysis and oxidative phosphorylation while inducing microglial phenotypic transition from the M1 to M2 polarization state, representing a sophisticated approach to neuroinflammation modulation [72].

Nutritional intervention strategies similarly have potential for regulating HK function [80]. The hydroxycinnamic acid components derived from olive oil significantly upregulate HK1 and phosphofructokinase M gene expression [80] through energy metabolism-related enzyme activity modulation, resulting in increased neuronal cell viability. These natural compounds offer multitarget engagement with minimal adverse effects, positioning them as promising candidates for translational neuroprotective strategies.

### 6.3 Integrated combination therapeutic strategies

Combination therapeutic approaches integrate direct and indirect strategies to achieve synergistic therapeutic efficacy while addressing multiple pathological processes simultaneously. These strategies represent the most comprehensive approach to HK-targeted neuro-therapeutics. The rationale for combination approaches stems from the multifactorial nature of neurodegeneration, where single-target interventions may be insufficient to address the complex pathological cascade.

#### 6.3.1 Inflammation-Metabolism Targets

The combination of anti-inflammatory agents with metabolic modulators has significant synergistic potential [15]. CY-09, a specific NLRP3 inflammasome inhibitor, combined with HK functional modulation, shows remarkable efficacy in AD models [74]. Research has revealed that CY-09 not only suppresses inflammasome activation but also significantly improves cerebral glucose metabolic function [74]. ¹^s^F-FDG PET imaging has demonstrated a marked improvement in whole-brain glucose utilization rates, accompanied by the restoration of HK1 and HK2 expression and their mitochondrial association [74].

This combination strategy’s advantage lies in simultaneously targeting inflammation and metabolism, two critical pathological processes in neurodegeneration, thereby achieving comprehensive neuroprotection through complementary mechanisms.

#### 6.3.2 Mitochondrial quality control integration

In PD treatment, the combined application of ROCK inhibitors with mitochondrial autophagy regulation shows promising potential [64]. ROCK inhibitors enhance PINK1/Parkin-mediated mitophagy while promoting HK2 recruitment to mitochondria, increasing Parkin localization, and facilitating damaged mitochondrial engulfment and degradation [64]. This strategy indirectly maintains HK function through improved mitochondrial quality control systems while directly promoting HK mitochondrial localization, establishing dual protective mechanisms.

#### 6.3.3 Growth Factors and Metabolic Signalling

Insulin signalling pathway modulation provides another critical direction for HK-targeted combination strategies. Studies have demonstrated [65] that insulin dose-dependently antagonizes the pathological effects induced by metabolic disorders through PI3K/AKT pathway activation, which inhibits NF-κB activation, reduces APP expression, and significantly decreases mitochondrial reactive oxygen species (ROS) levels. This protective effect is closely associated with HK function maintenance, as PI3K/AKT pathway activation promotes HK mitochondrial localization and activity regulation [65].

### 6.4 Precision Medicine and Personalized Approaches

The development of personalized therapeutic strategies based on precise HK expression level modulation represents a critical advancement in neurotherapeutics. Research reveals [30] a distinct dose-dependent relationship between HK2 expression levels and microglial activation states, where HK2 heterozygous deficiency confers protective effects, whereas complete deficiency produces detrimental outcomes. This finding indicates that therapeutic strategy design requires precise control of HK expression levels to avoid excessive activation or inhibition [30].

The implementation of gene therapy technologies for precise HK expression modulation, combined with real-time monitoring techniques for therapeutic efficacy assessment, may provide novel insights for personalized medicine approaches. Such precision-based strategies represent a paradigm shift toward individualized neurotherapeutics, where optimal HK expression thresholds can be tailored to specific pathological contexts, disease stages, genetic backgrounds, and individual patient metabolic profiles. The successful implementation of these precision medicine approaches requires sophisticated delivery technologies that can ensure accurate therapeutic delivery while maintaining the precision needed for personalized treatment protocols.

### 6.5 Advanced Delivery Systems and Drug Delivery Technologies

The successful clinical translation of HK-targeted therapeutics requires sophisticated delivery systems that can overcome multiple biological barriers while ensuring precise target engagement. Advanced delivery technologies represent a critical enabling component for the therapeutic strategies outlined in the previous sections.

#### 6.5.1 Mitochondrion-Targeted Delivery Systems

The development of mitochondrion-targeted delivery systems provides crucial technological support for HK-targeted therapeutics [81]. By encapsulating HK activators, stabilizers, or related peptide drugs within mitochondrion-targeted drug delivery carriers, precise subcellular drug delivery can be achieved, enhancing therapeutic efficiency while reducing systemic adverse effects. Triphenylphosphonium (TPP)-conjugated carriers demonstrate remarkable mitochondrial-targeting specificity, reaching 10-100-fold enrichment in mitochondrial compartments compared with their cytoplasmic distribution [82, 83]. These carriers successfully deliver HK-stabilizing peptides and small-molecule modulators directly to their sites of action, significantly enhancing therapeutic potency while minimizing off-target effects [82, 83]. The use of mitochondria-penetrating peptides (MPPs) is an alternative targeting approach that uses cell-penetrating sequences fused with mitochondrial targeting signals to achieve precise subcellular localization [84]. Recent developments in MPP technology have enabled the delivery of larger therapeutic payloads, including full-length HK proteins and regulatory enzymes, directly to mitochondrial matrices [85].

#### 6.5.2 Blood-brain barrier penetration techniques

Effective central nervous system (CNS) delivery represents a critical challenge for HK-targeted neurotherapeutics, which require specialized technologies to overcome blood-brain barrier restrictions. Focused ultrasound-mediated delivery enables transient, reversible blood-brain barrier opening, facilitating increased CNS penetration of HK-targeted therapeutics. Clinical studies have demonstrated the safe and effective delivery of large molecular weight compounds, including gene therapy vectors and protein therapeutics, with minimal adverse effects [86-88].

Receptor-mediated transcytosis systems utilize endogenous transport mechanisms to achieve CNS delivery. Transferrin receptor-targeted nanocarriers and insulin receptor-mediated delivery systems show particular promise for HK-targeted therapeutics, achieving therapeutic CNS concentrations while maintaining systemic safety profiles [89-91].

#### 6.5.3 Controlled Release and Sustained Delivery

Long-term therapeutic efficacy requires sustained drug delivery systems that maintain therapeutic concentrations over extended periods. Biodegradable polymeric systems provide controlled release kinetics tailored to specific therapeutic requirements. PLGA-based microspheres and nanoparticles enable the sustained release of HK modulators over weeks to months, reducing the dosing frequency while maintaining consistent therapeutic levels [92-94]. Implantable delivery devices offer the most sophisticated approach to long-term CNS drug delivery. These systems can be programmed for specific release profiles and refilled as needed, providing unprecedented control over therapeutic delivery while minimizing systemic exposure [95, 96].

#### 6.5.4 Combination delivery strategies

The integration of multiple therapeutic modalities requires sophisticated delivery systems capable of coordinated multidrug release. Sequential release systems enable time-controlled delivery of different therapeutic components, allowing for optimized therapeutic timing and synergistic effects [97, 98]. These systems can initially deliver anti-inflammatory agents, followed by metabolic modulators and neuroprotective compounds, according to predetermined schedules. Coencapsulation technologies allow the simultaneous delivery of multiple therapeutic agents while maintaining their individual stability and bioactivity [99, 100]. These approaches are particularly valuable for combination therapies that require precise stoichiometric ratios of different therapeutic components. This technological foundation positions HK-targeted therapeutics for successful clinical translation by addressing the critical challenges of target specificity, CNS delivery, and sustained therapeutic efficacy that are essential for treating chronic neurodegenerative conditions.

The therapeutic strategies presented in this chapter—from direct HK targeting to precision medicine approaches—represent a comprehensive toolkit for addressing HK dysfunction in neurodegeneration. The integration of these diverse approaches with advanced delivery technologies positions HK-targeted therapeutics as a promising new paradigm for neurodegenerative disease treatment, offering hope for conditions that have long resisted effective therapeutic intervention. As these strategies advance toward clinical application, their potential to transform the treatment landscape for neurodegenerative diseases becomes increasingly evident.

## 7. Critical Assessment: Challenges, Conflicting Evidence, and Future Directions

While the preclinical evidence supporting HK-targeted therapy in NDs is compelling, its translation from basic research to clinical application requires a rigorous and critical assessment. This chapter addresses the significant challenges related to efficacy and safety, discusses the technological innovations designed to overcome these hurdles, and outlines a strategic vision for future clinical translation.

### 7.1 Efficacy Limitations and Conflicting Evidence

A primary challenge lies in the nuanced and sometimes contradictory nature of HK's role. A balanced view must acknowledge findings that temper initial optimism. First, the neuroprotective efficacy of HK-targeting strategies is not universally consistent across different disease models. The benefits observed in acute injury models may not fully translate to chronic, slowly progressing neurodegenerative conditions. The specific underlying pathology—whether driven by amyloid, α-synuclein, or other factors—likely dictates the therapeutic ceiling of a purely metabolic intervention. This suggests that while HK is a critical node, its disruption may be a necessary but not always sufficient driver of neurodegeneration, making its modulation context-dependent. This complexity is exemplified by the dose-dependent effects observed in preclinical studies. Research has demonstrated a distinct relationship between HK2 expression and microglial activation, where heterozygous deficiency confers protection, but complete deficiency is detrimental [30]. This non-linear response underscores a critical point: therapeutic strategies require precise modulation, not simply maximal inhibition or activation, to avoid paradoxical outcomes.

### 7.2 Safety and Specificity: The Double-Edged Sword of a Central Metabolic Hub

The fundamental role of HK in cellular metabolism presents the most significant safety challenge: the risk of on-target toxicity in non-neuronal tissues. Evidence from the oncology field, where HK inhibitors are studied as anti-cancer agents, provides a cautionary tale. These studies highlight potential dose-limiting toxicities, including hematological and cardiac side effects, due to the systemic disruption of glycolysis [101-103]. This raises a crucial question for neurodegeneration: how can we achieve therapeutic benefit in the CNS while avoiding harm to other high-energy-demand organs^+^

This concern is particularly acute for the cardiovascular system. Cardiomyocytes are highly dependent on HK2 for energy metabolism, and HK2 plays a protective role in myocardial ischemia-reperfusion injury [20]. Consequently, systemic HK2 inhibition could compromise cardiac function, especially in an aging patient population with potential comorbidities. Similarly, altering HK function could affect hepatic glucose homeostasis and lipid metabolism, posing risks for patients with metabolic disorders [104]. The potential for HK2 overactivation to promote tumor cell proliferation also remains a critical long-term safety consideration [105, 106].

These risks are compounded by the tissue-specific expression of HK isoforms. HK1 is predominant in the brain, while HK2 is primary in muscle and adipose tissue [24, 33, 35, 68, 107, 108]. This distinction implies that non-specific HK modulators could produce a cascade of unpredictable systemic side effects while exerting their intended neuroprotective effects.

### 7.3 Overcoming Hurdles: Technological and Methodological Innovations

Despite these challenges, the scientific community is actively developing innovative strategies to enhance the safety and efficacy of HK-targeted therapies.

#### 7.3.1 Achieving CNS and Cell-Type Specificity

To address the critical issues of safety and off-target effects, research is focused on achieving precision at both the organ and cellular levels. A primary strategy is the development of blood-brain barrier (BBB)-targeted delivery technologies, which encapsulate HK modulators in carriers that utilize receptor-mediated transcytosis to significantly increase drug concentrations in the brain while minimizing peripheral exposure [109, 110]. To achieve an even finer level of control, cell-type-specific regulatory strategies are being advanced. By employing gene therapy vectors with cell-specific promoters, such as Synapsin for neurons or GFAP for astrocytes, HK modulation can be restricted to target cell populations [33, 111]. Furthermore, next-generation CRISPR technologies, including base and prime editing, offer the potential for precise and safe gene modifications without inducing double-strand breaks, promising an unparalleled degree of control over HK expression [112-114].

#### 7.3.2 Enhancing Preclinical Models and Monitoring

To better predict clinical outcomes, it is essential to overcome the limitations of traditional preclinical tools. The development of advanced models, such as three-dimensional brain organoids and organ-on-a-chip platforms, represents a significant step forward, moving beyond simplistic 2D cell cultures to better simulate complex neuron-glia interactions and the physiological in vivo environment [115, 116]. Complementing these improved models, new monitoring technologies provide unprecedented insights into HK dynamics. For instance, FRET-based biosensors enable real-time tracking of intracellular HK activity, while PET imaging with specific tracers allows for noninvasive monitoring of HK function in living organisms, providing crucial biomarkers for diagnosis and therapeutic response [117, 118]. The vast amount of data generated by these platforms can then be harnessed through high-throughput analytical techniques. Single-cell omics technologies, for example, are revealing the cell-specific roles of HK in disease with remarkable resolution [16]. Moreover, integrating these large-scale datasets with artificial intelligence and machine learning can accelerate the identification of novel biomarkers and streamline the computer-aided design of new drug molecules [119, 120].

### 7.4 From Bench to Bedside: Clinical Translation and Strategic Vision

The ultimate success of HK-targeted therapies hinges on a clear and strategic pathway to the clinic.

#### 7.4.1 The Imperative for Precision and Combination Therapies

A one-size-fits-all approach is unlikely to succeed given the heterogeneity of NDs. The future therefore lies in precision medicine, which aims to stratify patients based on their unique biological profiles. Integrating genomic (e.g., APOE genotype), metabolic (e.g., PET imaging), and other biomarker data will be essential for identifying patient subgroups most likely to benefit from a specific HK-targeted intervention [11, 21, 121, 122]. Beyond patient stratification, the multifactorial nature of these diseases suggests that single-target therapies may be insufficient. Consequently, developing combination strategies that pair HK-targeted therapy with other approaches—such as anti-inflammatory, antioxidant, or proteostasis-enhancing treatments—may produce synergistic effects and represent a more robust therapeutic paradigm [15, 18, 60].

#### 7.4.2 Navigating the Clinical and Regulatory Landscape

The path to approval is fraught with practical challenges. Navigating this landscape begins with addressing complex regulatory hurdles, as HK-targeted therapies span a wide range from small molecules to gene therapies, each with distinct requirements. Once a regulatory path is defined, the design of clinical trials presents its own set of challenges, particularly given the slow progression of NDs and patient heterogeneity, which makes defining meaningful endpoints difficult. The development of validated biomarkers for target engagement and therapeutic response is therefore crucial for conducting efficient and conclusive trials. Finally, beyond the scientific and regulatory complexities, significant practical hurdles related to cost-effectiveness, scalable manufacturing, and equitable market access must be overcome to ensure these advanced therapies can ultimately reach the patients who need them.

#### 7.4.3 Long-term Vision and Concluding Remarks

The convergence of advancing technology and deepening mechanistic understanding positions HK-targeted therapy as a transformative approach for NDs. The long-term vision is a precision medicine ecosystem where real-time metabolic monitoring guides adaptive, personalized treatments, potentially even as preventive interventions in at-risk populations.

In summary, while the road to clinical application is challenging and requires a clear-eyed view of the existing limitations and conflicting evidence, the potential of HK as a therapeutic nexus remains immense. Acknowledging the gaps in our knowledge, including a likely publication bias against null findings, is essential for fostering rigorous and reproducible science. Through multidisciplinary collaboration and continued innovation, HK-targeted therapies hold the promise of providing new hope for patients with neurodegenerative diseases, marking a significant advance in the field of metabolic neuroscience.
